# Kentucky Institution for the Blind

**Published:** 1847-04

**Authors:** 


### THE WESTERN JOURNAL.

New Series.—Vol. VII.—No. IV.

LOUISVILLE, APRIL 1, 1847.

### KENTUCKY INSTITUTION FOR THE BLIND.

We have lately received the fifth annual report to the Legislature of the board of visitors of this interesting school, established a few years ago by the Commonwealth of Kentucky in connection with many benevolent citizens of Louisville. The number of pupils at the close of the late calendar year was 31, of whom 13 were admitted during the year, six or seven having been discharged through the same period. Of the latter the board remark—

"Several of the pupils who have been discharged in the last year had been in the school but a short time, and from age or want of capacity gave little promise of being much benefited by a longer continuance in the institution. And here it may be well to repeat what has been heretofore stated, that the institution is strictly a school for the education of blind youth of good mental capacity, and not an asylum for the old or imbecile, for whom suitable asylums should be and are elsewhere provided, as they can derive but little advantage from associating with the young and active, while the latter may suffer immensely from such association. We therefore urge upon all friends of the blind, who may apply for the admission of pupils into the institution, the importance of making known as far as may be the mental capacity, character and habits of each candidate. By care in this respect, the expenses of fitting out and transporting children to the institution may perhaps in some instances be avoided. Nor can we too earnestly press the importance of sending pupils at an early age. The circumstances by which the blind are too frequently surrounded at home are exceedingly unfavorable to mental, moral and physical improvement; and the most serious difficulties with which the officers of our institution have to contend had their origin in the early homes of the pupils, where, untaught if not uncared for, they passed their childhood and in some cases their youth. The nobler powers, neglected, go to decay, while the lower faculties, without culture, increase in strength; the proper balance of the mind, destroyed, is with the utmost difficulty restored; and not only the school days but the entire life may be wasted in unavailing attempts to restore to their place, passion and reason and conscience. If the consequences are confined to the unfortunate individuals, the evil is less; but placed in a school, they exert a most unfavorable influence on all their fellow students. A proper regard, therefore, for the best interests of the pupils and the institution makes it incumbent on us to scrutinize very carefully the character of every candidate for admission."

We have extracted this paragraph, in the hope that our brethren in the adjoining States (who should consider themselves in some degree the natural guardians of the blind,) will do what may be practicable in the case—that is, give to parents such advice as will prevent those who are unfit to become scholars from being sent. At the same time we would exhort them to search out others, and facilitate their being sent to the institution. Every part of the country contains blind children whose parents have not even heard of the school, or if they have, are incredulous as to the advantages it can impart. From the want of early medical attention, the children of the poor are peculiarly apt to lose their sight when attacked with our endemic ophthalmia. The report now before us affords indubitable evidence of this fact. It states that of the thirty-one pupils in the school on the 19th of January last, twenty-one were beneficiaries of Kentucky, five of the State of Indiana, and one of a benevolent society in New Orleans, leaving only *four* pay pupils. Again we say to all our readers, remember the blind children of your neighborhoods, and use your influence to have them sent to the Kentucky or some other institution, where by cultivation of the senses of touch and hearing, their intellectual, moral and physical capabilities may be improved, and their happiness and usefulness increased.

